# Changes in the Gut Microbiota after the Use of Herbal Medicines in Overweight and Obese Individuals: A Systematic Review

**DOI:** 10.3390/nu15092203

**Published:** 2023-05-05

**Authors:** Miguel Huang, Cláudia dos Santos Cople-Rodrigues, Dan L. Waitzberg, Ilanna Marques Gomes da Rocha, Cintia Chaves Curioni

**Affiliations:** 1 Postgraduate Program in Food, Nutrition and Health (PPG-ANS), University of State of Rio de Janeiro, Rio de Janeiro 20550-170, Brazil; miguelhuang1973@gmail.com; 2 Department of Applied Nutrition, University of State of Rio de Janeiro, Rio de Janeiro 20550-170, Brazil; claudiacople@gmail.com; 3 Department of Gastroenterology, Faculdade de Medicina, LIM–35, Hospital das Clinicas HCFMUSP, School of Medicine, Universidade de São Paulo, São Paulo 05508-090, Brazil; dan.waitzberg@gmail.com (D.L.W.); ilanna.marques@gmail.com (I.M.G.d.R.); 4 Department of Nutrition in Public Health, University of State of Rio de Janeiro, Rio de Janeiro 20550-170, Brazil

**Keywords:** gut microbiota, herbal medicine, obesity, overweight, dysbiosis

## Abstract

Background: Herbal medicine is a low-cost treatment and has been increasingly applied in obesity treatment. Gut microbiota (GM) is strongly associated with obesity pathogenesis. Methods: We conducted a systematic review guided by the question: “Does the use of herbal medicine change the GM composition in obese individuals?” Randomized clinical trials with obese individuals assessing the effects of herbal medicine intervention in GM were retrieved from the Medline, Embase, Scopus, Web of Science, and Cochrane Library databases, including the Cochrane Controlled Trials Register. Two reviewers independently extracted data using standardized piloted data extraction forms and assessed the study-level risk of bias using an Excel template of the Cochrane “Risk of bias” tool 2—RoB 2. Results: We identified 1094 articles in the databases. After removing duplicates and reading the title and abstract, 14 publications were fully evaluated, of which seven publications from six studies were considered eligible. The herbs analyzed were *Moringa oleifera*, *Punica granatum*, *Scutellaria baicalensis*, *Schisandra chinensis*, W-LHIT and WCBE. The analysis showed that *Schisandra chinensis* and *Scutellaria baicalensis* had significant effects on weight loss herbal intervention therapy composed by five Chinese herbal medicines *Ganoderma lucidum*, *Coptis chinensis*, *Astragalus membranaceus*, *Nelumbo nucifera gaertn*, and *Fructus aurantii* (W-LHIT) and white common bean extract (WCBE) on GM, but no significant changes in anthropometry and laboratory biomarkers. Conclusions: Herbal medicine modulates GM and is associated with increased genera in obese individuals.

## 1. Introduction

Obesity is currently a metabolic disease associated with an increased risk for the emergence of other diseases, such as type 2 diabetes mellitus and cardiovascular disease, leading causes of death worldwide [1]. In 2016, the World Health Organization [2] estimated that more than 1.9 billion adults were overweight.

The primary strategies for obesity treatment encourage changes in lifestyle, such as shifting to a healthy eating pattern, rich in natura or minimally processed foods, and regular physical exercise, among others. There is a thriving interest in medicinal plants for the prevention and treatment of different diseases, including endocrine ones, even though the Eastern world has been using them for centuries [3]. In this context, herbal medicine can play an important role in obesity treatment through mechanisms, such as stimulating thermogenesis, inhibiting pancreatic lipase activity, reducing food intake, reducing fat absorption, increasing lipolysis and decreasing lipogenesis [4]. These actions are associated with the presence of secondary metabolites featured in plants, such as flavonoids, saponins, and essential oils, among others, which trigger important physiological changes [5]. In this way. Thus, herbal medicine shows conceivable therapeutic benefits toward metabolic disorders [6], opening other paths through the treatment of obesity.

Human GM has been included in the list of factors studied in relation to obesity, as well as other comorbidities [7]. Gut microorganisms, including bacteria, archaea, fungi, and viruses, may impact the development of metabolic disorders in different ways, such as by altering dietary energy production, causing imbalance in the composition of adipose tissue, affecting inflammatory processes, causing intestinal barrier disruption, as well as affecting the regulation of appetite through the gut–brain axis [8].

The interaction between herbal medicine and GM can occur in multiple ways. One mechanism is by altering the composition and function of microorganisms, specifically bacteria [9]. Another mechanism involves the action of metabolites derived from medicinal plants during the metabolic process in the body [10]. Hence, the modulation of the GM through herbal medicines [11] represents a novel frontier for developing medicines or drugs to treat obesity.

Therefore, we conducted a systematic review to assess the effects of herbal medicine on the GM of overweight and obese individuals. By examining the potential effects of herbal medicine on the GM of this population, we can gain insight into new approaches for reversing obesogenic processes and potentially unraveling new treatments.

## 2. Materials and Methods

### 2.1. The Materials

#### Registration and Protocol

We registered the protocol for this systematic review in the international prospective register of systematic reviews—PROSPERO (CRD42022298264)—and reported our findings in accordance with the Preferred Reporting Items for Systematic Reviews and Meta-Analyses (PRISMA) guidelines.

### 2.2. Review Question

Does the use of herbal medicine change the GM composition in obese individuals?

Criteria for considering studies for this review.

The inclusion criteria followed the constitutive elements of the acronym PICOT, and are as follows:

Participants

Individuals over 18 years old, of both genders, with a diagnosis of overweight or obesity (body mass index—BMI—above 25 kg/m^2^).

Intervention

Any herbal agent used alone or in combination with other herbal medicines or other forms of prescription for treating obesity and its possible changes in the GM.

Comparison

Placebo, another herbal agent, or another intervention.

Outcome(s)

Primary: changes in the GM, including reduction in pathogenic species, the proportion of phyla Firmicutes and Bacteroidetes, alpha and beta diversity (Chao, Simpson, and Shannon index), etc.

Secondary: anthropometric measurements and main laboratory biomarkers linked to obesity-derived comorbidities. Adverse effects were also investigated since the dosage levels and pharmaceutical forms of herbal medicines can increase toxicity or cause synergistic reactions.

Types of study that were included

This study included published randomized clinical trials.

Exclusion Criteria

Interventions with isolated constituents of plant origin (not the whole plant), vegetable oils, and other dietary supplements (such as fiber or protein); studies involving patients who have undergone bariatric surgery or other surgical procedures on the gastrointestinal tract were excluded from the analysis.

### 2.3. Search Methods for Identification of Studies

The identification of studies was performed in Medline, Embase, Scopus, Web of Science, and Cochrane Library databases, including the Cochrane Controlled Trials Register, from inception to April 2023. In addition, the reference lists of the included studies and reviews were searched to identify any additional relevant studies. There were no restrictions applied for language or date of publication. For the search strategy, a combination of MeSH Terms and keywords was used; terms related to phytotherapy or herbal medicine AND terms related to microbiota AND terms related to obesity. The complete search strategy is described in Supplementary Material S1.

### 2.4. Study Selection

The results were transferred to the Rayyan QCRI, a systematic review web app. After the removal of duplicate documents, two researchers (MH, CC) independently screened the records’ titles and abstracts. The full texts of potentially eligible records were reexamined and independently screened again (MH, CC) to ensure inclusion. Disagreements and discrepancies were solved through discussion by all the researchers.

### 2.5. Data Extraction and Risk of Bias Assessment

Two reviewers (MH, CC) independently extracted data using standardized piloted data extraction forms, and assessed the study-level risk of bias using an Excel template of the Cochrane “Risk of bias” tool 2—RoB 2.

For each trial, we extracted the following data: identification of study, country, type of study, number of participants, randomization process, blinding process, and characteristics of the enrolled sample (age and sex); characteristics of the intervention (type, method, dosage, and duration of the intervention) and attributes of control/placebo; outcomes assessed and results; information on conflict of interest; and adverse effects of the intervention. When information regarding any of the above was unclear or incomplete, we attempted to contact the authors of the original reports to request further details by email.

RoB 2 is structured into five domains: (1) Randomization process; (1.1) Timing of identification or recruitment of participants in a cluster-randomized trial (only for cluster trials); (2) Deviations from intended interventions; (3) Missing outcome data; (4) Measurement of the outcome; and (5) Selection of the reported result. For each domain, responses to the signaling questions were categorized as “yes”, “probably yes”, “probably no”, “no”, or “no information”. A proposed algorithm was then used to determine the judgment about the risk of bias for each domain, which was classified as either “low risk of bias”, “some concerns”, or “high risk of bias”.

After this step, we judged the overall risk of bias of the study according to whether the trial had low-risk bias (in all domains); some concerns (in at least one domain, but not with high-risk bias in any domain); or high-risk bias (in at least one domain or some concerns in multiple domains). The RoB 2 plot was generated by risk-of-bias visualization (robvis) [12].

### 2.6. Data Synthesis

Data analysis utilized a narrative approach to summarize the effect of the interventions. Due to the high degree of heterogeneity among the studies, it was not feasible to conduct a quantitative synthesis.

In order to standardize the results, the difference of means for the intervention group and the control group between the beginning and end of the follow-up was calculated for continuous data of both biochemical and anthropometric variables (i.e., mean change of the variable: final mean − mean at baseline), as well as the difference in standard deviation (SD) over the same period. The treatment effect was estimated by calculating the mean difference between the intervention and control groups, along with their respective 95% confidence intervals. This analysis was performed automatically using the Review Manager software (RevMan 5.4.1).

## 3. Results

### 3.1. Results of the Search

In the preliminary stage of searching for articles, we retrieved 1094 records from the databases. After removing the duplicates, 790 titles and abstracts were screened in accordance with the inclusion criteria. Therefore, 14 publications were fully evaluated, 7 of which were excluded.

**Figure 1:**
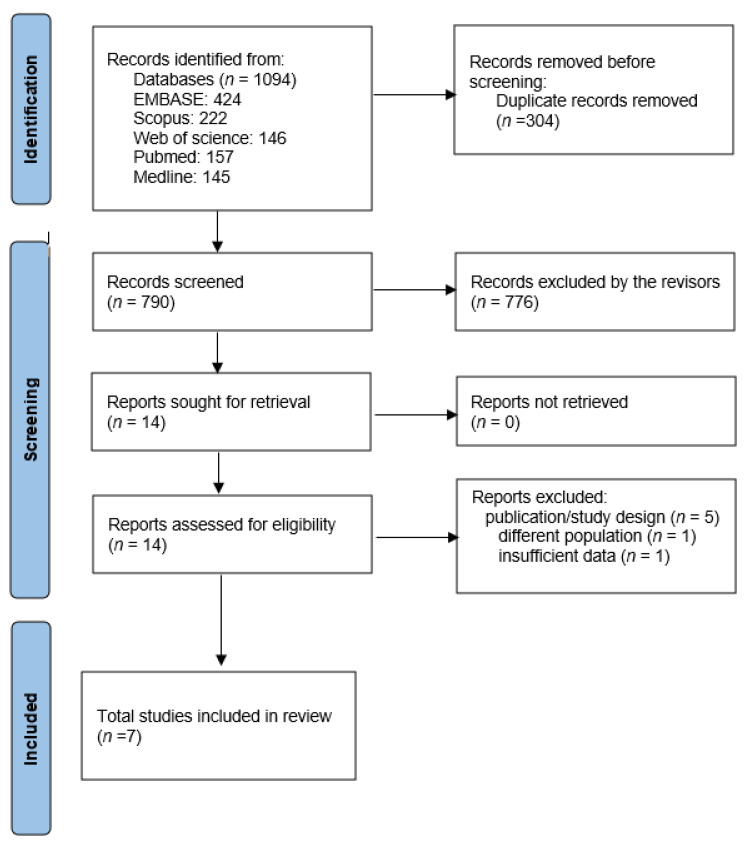
PRISMA study flow diagram for search up to April 2023.

There was an attempt to contact the authors of one study with insufficient data on the GM results. However, because we received no response, this article was excluded [13]. Finally, seven publications from six studies were included in the review. Figure 1 shows an overview of the screening procedure.

### 3.2. Study Characteristics

Five studies were double-blind, parallel randomized clinical trials, and the other two had a crossover design [14,15,16,17]. They were published between 2015/2023 and carried out in three countries: China, South Korea, and Spain. The individuals’ ages ranged from 20 to 75 years old. One study [17] included only females, while the others encompassed both sexes. Two studies were limited to the overweight population. It is noteworthy that two complementary publications of the same study were found and were considered as one [18,19].

The herbal medicines studied were *Moringa oleifera* [16], *Punica granatum* [18,19], *Scutellaria baicalensis* [20], *Schisandra chinensis* [17], and weight loss herbal intervention therapy consisting of five Chinese herbal medicines: *Ganoderma lucidum*, *Coptis chinensis*, *Astragalus membranaceus*, *Nelumbo nucifera gaertn*, and *Fructus aurantii* (W-LHIT) [14], and white common bean extract (WCBE) [15].

In the study by Goméz-Martinéz et al. [16], *Moringa oleifera* was offered in 400 mg/capsules. The participants were instructed to take two capsules before the three main meals (breakfast, lunch, and dinner) for 12 weeks. Morbid obesity (body mass index, BMI > 35 kg/m^2^) was used as an exclusion criterion.

The study by González-Sarrías et al. [18,19] has a crossover design. Participants received a daily dose of 450 mg of *Punica granatum* for three weeks, followed by a three-week washout period, and then another daily dose of 1800 mg for three weeks. Although the clinical trials treated the same population, they differ in their approaches and research methods regarding GM, being complementary in their results.

In the Shin et al. study [20], *Scutellaria baicalensis* was tested in association with the use of metformin in a crossover trial. The dosage given was 3.520 mg/day for eight weeks, followed by a wash-out period of four weeks, and a further eight weeks with a placebo. In both periods, the dosage of metformin was maintained according to the previous medical prescription of each participant. In the study conducted by Song et al. [17], participants consumed *Schisandra chinensis* in liquid form, provided in bags of 100 mL (two units) containing approximately 6700 mg of dry extract per day for a duration of eight weeks.

Extraction from white common beans (*Phaseolus vulgaris*) was applied in the study of Feng [15], being offered in doses of 1.5 g for each meal daily over the course of 4 months.

The other Chinese clinical trial by Cao [14] prescribed capsules composed of five herbs (*Ganoderma lucidum*, *Coptis chinensis*, *Astragalus membranaceus*, *Nelumbo nucifera gaertn*, and *Fructus aurantii*). The intervention group dosage was based on individual body weight, varying from 9 to 15 capsules a day for two months. Table 1 describes the details of each study.

### 3.3. Gut microbiota

GM research was highlighted as the main outcome in five articles [14,15,18,19,20], while two others placed it as a secondary outcome [16,17]. Table 2 describes the main finding of microbiota analysis.

Clinical trials with W-LHIT [14], WCBE [15], *Punica granatum* [19], *Scutellaria baicalensis* [20], and *Moringa oleifera* [16] used 16srRNA metagenomic technology for the analysis of GM. The other clinical trials [17,18] collected data using the qPCR technique.

The study by González-Sarrías consisted of two separate publications, each utilizing different approaches for the analysis of the GM. In the first publication, which employed the qPCR technique [18], there was a significant increase in the genera *Gordonibacter*, *Bacteroides*, and *Escherichia coli*, alongside a reduction in lactic acid bacteria. The research on the GM has focused on bacteria involved in the conversion of secondary metabolites to urolithin, narrowing its spectrum of bacterial analysis.

The other publication [19] sought to observe broader alterations in the GM with the use of *Punica granatum*, in addition to verifying changes in the endotoxemia. For the interventional group, at the phylum level, there was an increase in *Bacteroidetes* and a decrease in *Firmicutes*. There was also an increase in *Bacteroides* and *Faecalibacterium* genera, with a reduction in *Romboutsia*, *Anaerostipes*, *Dorea*, and *Clostridium sensu stricto*. At the family level, there was an increase in *Bacteroidaceae* and *Porphyromonadaceae*, and a reduction in *Peptostreptococcaceae*, *Clostridiaceae*, and *Coriobacteriaceae*.

The same phylum change was also observed in the clinical trial conducted by Song using *Schisandra Chinensis* as an intervention [17], which led to a subsequent reduction in the *Firmicutes/Bacteroidetes* ratio. At the genus level, the intervention group showed an increase in *Akkermansia*, *Roseburia*, *Bacteroides*, *Prevotella*, and *Bifidobacterium*, while only *Ruminococcus* exhibited a decrease compared to the placebo group.

In the trial that combined *Scutellaria baicalensis* with metformin [20], there was an increase in the abundance of *Megamonas*, *Mobilitalea*, *Acetivibrio_g1*, *Lactobacillus*, and *Akkermansia*. Conversely, a decrease in *Clostridium_g23*, *Oscillibacter*, *Alloprevotella*, and *Bifidobacterium* was observed. The *Weissella* genus did not show any significant change in either the treatment or control groups.

The use of *Moringa oleifera* [16] as a strategy for glycemic control did not find changes in the GM composition for Bacteroides, *Blautia coccoides*, *Eubacterium rectale*, *Clostridium cluster IV*, *Bifidobacterium* spp., *Lactobacillus* spp., and *Enterobacteriaceae*. After the consumption of Moringa oleifera, the only bacterial group to show an increase was *Enterococcus* spp. However, there were no significant changes observed in the levels of *Faecalibacterium prausnitzii* and *Akkermansia muciniphila*, which are considered bacterial species markers of gut health.

By analyzing the GM using WCBE [15], we observed an enrichment of SCFA-producing bacteria, notably the genus *Bifidobacterium*, and a reduction in other genera that appear in the taxonomic level of the *Enterobacteriaceae* family, which contains several opportunistic pathogens.

Diversity was evaluated in four trials [14,15,19,20], but without significant changes, except for a slight increase found at Cao [14].

### 3.4. Anthropometric and Biomarkers Data

As a secondary objective of this systematic review, we sought to identify outcomes related to the anthropometry and laboratory biomarkers of the selected clinical trials. As well as the GM data, the articles presented varied data. These divergences result in comparative limitations. Table 3 presents the results for the parameters evaluated. When comparing the effects of the evaluated herbal medicines, no significant changes were observed in any variable.

The study by González-Sarrías [18] grouped the results of the surveyed population by type of urolithin (UM-A, UM-B, UM-0) presented in the urine after the clinical trial. Thus, it was not possible to establish a comparison between the intervention group and the control group for the secondary objectives of this review.

Only two studies reported adverse effects resulting from the consumption of herbal medicines. In the clinical trial conducted by Shin et al. [20] using *Scutellaria baicalensis*, one of the participants in the intervention group reported epigastric pain. The study by Cao et al. [14] described two subjects with slight gastrointestinal reactions in the treatment group.

### 3.5. Risk of Bias in Included Studies

**Figure 2:**
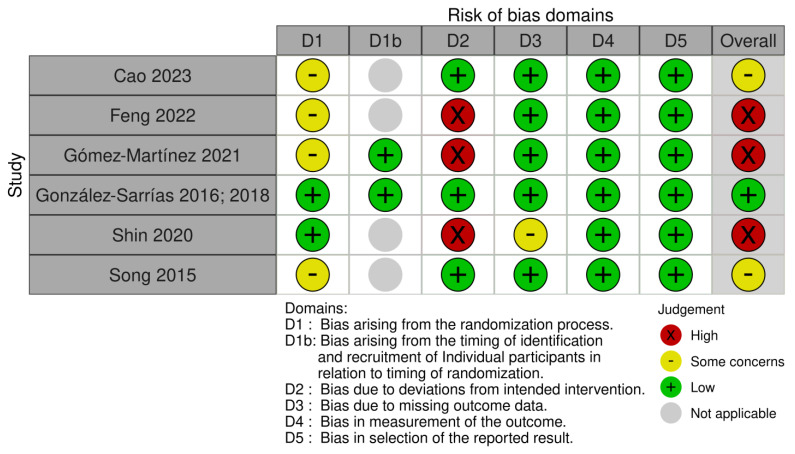
Risk of bias of included studies. Cao 2023 [14]; Feng 2022 [15]; Gómez-Martínez [16]; González-Sarrías [18,19]; Shin [20]; Song 2015 [17].

The risk of bias assessment of the selected articles, including all domain judgments, is shown in Figure 2. Only the study by González-Sarrias was judged as low risk of bias [18,19]. Three studies were judged to be at high risk of bias [15,16,20] since they did not perform an intention-to-treat analysis and showed a loss of the surveyed population by more than 5%. In addition, Cao [14] and Song [17] did not elaborate on the randomization process and were judged as “some concerns”.

## 4. Discussion

The GM has been a growing research topic in the health area for its role in influencing diseases such as obesity. Moreover, herbal medicine presents itself as a treatment with fewer deleterious side effects compared to other types of intervention in the fight against excess weight. Knowing and understanding how herbal medicines can modulate the GM may open new perspectives of adjuvant action for overweight patients.

This systematic review selected six randomized clinical trials that evaluated the use of herbal medicines in the human microbiota involving 296 overweight patients, aged between 18 and 75 years. Although some positive effects have been verified, the evidence still presents gaps.

Changes in diversity were evaluated in four out of the six included studies, with no change for this indicator after the herbal intervention [14,15,19,20]. Diversity reduction, considered an indicator of dysbiosis, has been associated with different chronic conditions, such as obesity and type 2 diabetes [21]. The studies involving dietary interventions were able to establish a relationship between greater bacterial diversity with health benefits for overweight and obese patients [22,23,24]. The present review did not find significant changes in bacterial diversity from the intervention of selected herbal medicines, either due to a lack of data or results of low relevance.

At the beginning of the study of the microbiota associated with obesity, it was presumed that adequacy in the ratio between *Firmicutes*/*Bacteroidetes*, with proportion values ranging from 0.7 to 1.0, could also be used as a sign of improvement in the GM profile of obese individuals. Based on that proposition, three studies [14,17,19] brought up the *Firmicutes*/*Bacteroidetes* ratio in the description of their results. Two studies demonstrated a reduction [17,19] and one study [14] showed an increase in this ratio. However, no correlation was found between these changes and the reduction in the anthropometric values or biomarkers observed in the intervention group of the aforementioned studies.

Two clinical trials that analyzed the taxonomic levels of the family, genus, and species showed clinically insignificant changes [19] or biases related to drug–herbal drug interaction [20]. Recent findings on the complexity of the gut microbial ecosystem suggest that a higher relative abundance of the phylum *Firmicutes* and a lower relative abundance of Bacteroidetes in obese individuals do not always reflect a common pattern for the genera belonging to these phyla since several genera of the same phylum can be found to a greater or lesser extent in obese individuals [25,26]. In addition, bacterial interactions, such as commensalism, mutualism, amensalism, and the symbiotic relationship with their host increasingly reveal the role of each bacterium in the context of the “obese” microbiota profile, extrapolating the stratification by taxonomic level [27].

The genus *Akkermansia*, belonging to the phylum *Verrucomicrobia*, can colonize the mucosal layer in the intestines and modulate basal metabolism. The association between *Akkermansia* and obesity is consistent, with several studies demonstrating the bacteria adjuvant potential to control low-grade chronic inflammation and obesity [28]. Due to its significant role in studies of GM, all selected clinical trials investigated its abundance. *Akkermansia* enrichment has been reported after intervention with W-LHIT [14], *Scutellaria baicalensis* [20], and *Schisandra chinensis* [17].

Another genus evaluated in the selected articles was *Bifidobacterium*, associated with the production of SCFAs acetate, propionate, and butyrate. Studies relate the presence of this genus to the profile of eutrophic individuals [29,30]. Among the findings, we emphasize the enrichment of *Bifidobacterium* in the consumption of W-LHIT [14], *Schisandra chinensis* [17], and *Punica granatum* [19], which could guarantee benefits for the balance of the GM.

The selected articles did not report significant effects on the biomarkers and anthropometric data measured during the clinical trials, except for one study. However, another systematic review examining the use of herbal medicines for weight loss failed to provide sufficient evidence of efficacy in randomized controlled trials [31]. In contrast, a systematic review with a meta-analysis [4] showed significant effects on some biomarkers and anthropometric data.

### Limitations

The clinical trial with *Scutellaria baicalensis* was associated with the use of metformin [20], a drug of the biguanide type, indicated as a first-line treatment in individuals diagnosed with type 2 diabetes. Studies prove its modulating role in the GM, favoring the increase in bacterial populations such as *Akkermansia* and *Lactobacillus* [32,33]. This interaction may mask the effect of the herbal medicine on changes in the GM, considering that there is no information about the amount and time of metformin use in this clinical trial.

Methodological differences may have contributed to inconsistencies in bacterial counts. Refrigeration at 4 °C was associated with no significant change in fecal microbiota diversity or composition. On the other hand, samples stored using other conditions, such as primers, showed substantial divergence compared to control samples under low refrigeration [34,35]. The fecal sample goes through several steps until it is translated into clinical interpretation through the taxonomic identification of the bacteria [36].

In addition, the small sample size in all included studies and the impossibility of carrying out the meta-analysis due to these differences are highlighted. It was also observed that the herbs were used with different duration times, less than a period of 8 weeks. Clinical trials with short-term interventions show transient changes in the GM that do not last [37]. Obesity is a complex disease in which, in most cases, a sustainable long-term intervention is required to achieve weight loss results [38].

The GM undergoes changes due to several factors, whether extrinsic or intrinsic. Diet is the main modulator, capable of changing the composition of the bacteria that make up the GM in a short time [39]. The studies did not specify the diet composition of the participants throughout the trial period. Macronutrients and micronutrients can modulate certain groups of bacteria [8], changing the data found in the analysis of clinical trials.

This is the first systematic review to evaluate changes in the GM of overweight and obese populations from randomized clinical trials using herbal medicines in the intervention. Despite the vast scientific literature in the field of GM since the elaboration of the MetaHIT (Metagenomics of the Human Intestinal Tract) [40], generating a valuable database on the GM, randomized clinical trials are still scarce, whether due to ethical issues or limitations in controlling confounding factors [41]. The study was carefully conducted following current protocols and methodological recommendations.

## 5. Conclusions

The present systematic review showed that despite the slight changes in the GM of overweight individuals after the use of herbal medicines, there is still an incipient consensus regarding the effects of the use of herbal medicine. The binomial time and dosage in clinical trials involving herbal medicine in obesity treatments are diverse, and parameters must be established that allow a greater degree of comparability on their effects on the GM.

Integration of the GM and metabolomics opens a promising path for personalized modulation. Metabotyping (grouping of individuals with comparable metabolic/phenotypic profiles) effects the GM according to the individual degree of herbal medicine metabolism. High heterogeneity in microbial population changes within intervention groups in clinical trials was found in this study. Thus, these facts may indicate that personalized nutritional recommendations can pave the way for different doses of herbal medicine as an adjuvant treatment for obesity and its comorbidities.

Research standards must be established to allow statistical analysis of the diversity of the GM and its relationship with diseases and conditions such as obesity. Future randomized clinical trials promoting more stratified techniques may contribute toward data robustness and allow meta-analyses to obtain quantitative results of the effects of herbal medicine on the GM of obese and overweight patients.

## Figures and Tables

**Table 1:** Characteristics of the studies included in the systematic review.

Author, Country	Age (Years)	BMI (kg/m^2^)	Gender	Comorbities	nB/N Endpoint (% Dropout)	Intervention/Control	Dosage	Duration
Cao [14] China	18 to 60	32.25 ± 1.4 (intervention) 34.04 ± 2.5 (control)	M: 24; F: 13	-	37/40 (7.5%)	W-LHIT	9 to 15 capsules/day	2 months
Feng [15] China	35 to 75	27.9 ± 0.4 (intervention) 25.1 ± 0.5 (control)	M: 33; F: 50	Diabetes T2D	83/96 (13.5%)	WCBE	1.5 g before each meal/day	2 months
Gómez-Martínez [16] Spain	45 to 70	28.6 ± 3.8 (intervention) 29.4 ± 4.0 (control)	M: 29; F: 36	Prediabetes	73/65 (11.0%)	*Moringa* *oleifera*	2.4 g/day	12 weeks
González-Sarrías, [18,19] Spain	>40	28.5 ± 1.1 overweight 33.2 ± 3.3 obese	M: 32; F: 17	-	50/49 (2.0%)	*Punica* *granatum*	0.45 g/day (3 weeks) 1.8 g/day (3 weeks)	24 weeks (3 weeks of wash out between dosage)
Shin [20] South Korea	20 to 75	25.62 ± 0.64 (intervention) 25.69 ± 0.62 (control)	F and M	Diabetes T2D	17/12 (29.4%)	*Scutellaria* *baicalensis*	3.52 g/day	8 weeks (4 weeks of wash out)
Song [17] South Korea	25 to 45	29.99 ± 4.27 (intervention) 28.78 ± 3.47 (control)	F	-	40/28 (30.0%)	*Schisandra chinensis*	6.7 g/day	12 weeks

T2D: type 2 diabetes; BMI: body mass index; nB/N: baseline subjects/subjects finished; WCBE: white common bean extract; W-LHIT: weight loss herbal intervention therapy; F: female; M: male

**Table 2:** Changes of the GM following the administration of herbal medicines in overweight and obese individuals.

Article	Intervention	Microbiota Analysis Method	GM Changes
Cao [14] China	9 to 15 capsules W-LHIT/day	16S rRNA	Increase in phylum *Verrucomicrobia*, and decrease in phylum *Proteobacteria*. Increase in genera *Akkermansia* and *Enterococcus.* Decrease in species *Eubacterium rectale*, *Haemophilus parainfluenzae*, and *Faecalibacterium prausnitzii*.
Feng [15] China	1.5 g WBCE before each meal/day	16S rRNA	Increase in genera *Anaerostipes*, *Bifidobacterium*, *Faecalibacterium*, *Faecalitalea*, *Lactobacillus*, and *Romboutsia*, and decrease in genera *Adlercreutzia*, *Citrobacter*, *Cronobacter*, *Enterobacteriaceae*, *Fusobacterium*, *Klebsiella*, and *Weissella*.
Gómez-Martínez [16]	2.4 g dry extract MO/day 12 weeks	16S rRNA	No significant change in *Clostridium cluster IV* and in genera *Bifidobacterium* and *Lactobacillus*. No significant change in species *Blautia coccoides*, *Eubacterium rectale*, *Faecalibacterium prausnitzii*, and *Akkermansia muciniphila*.
González-Sarrías [18]	0.45 g dry extract PG/day 3 weeks 1.8 g dry extract PG/day 3 weeks	real-time qPCR	Increase in genera *Gordonibacter* and *Bacteroides*. Increase in species *Escherichia coli*.
González-Sarrías [19]	0.45 g dry extract PG/day 3 weeks 1.8 g dry extract PG/day 3 weeks	16S rRNA	Increase in phylum *Bacteroidetes*, and decrease in phylum *Firmicutes*. Increase in families *Bacteroidaceae* and *Porphyromonadaceae*, and decrease in families *Peptostreptococcaceae*, *Clostridiaceae*, *and Coriobacteriaceae*. Increase in genera *Bacteroides* and *Faecalibacterium*, and decrease in genera *Romboutsia*, *Anaerostipes*, *Dorea*, and *Clostridium sensu stricto*. No significant changes in bacterial diversity.
Shin [20]	3.52 g dry extract SB/day 8 weeks	16SrRNA	Increase in genera *Lactobacillus*, *Weissella*, and *Akkermansia.* No significant changes in bacterial diversity.
Song [17]	6.7 g dry extract SC/day 12 weeks	qPCR	Increase in phylum *Bacteroidetes*, and decrease in phylum *Firmicutes*. Increase in genera *Akkermansia*, *Roseburia*, *Bacteroides*, *Prevotella*, and *Bifidobacterium*; decrease in genus *Ruminococcus*.

WCBE: white common bean extract; W-LHIT: weight loss herbal intervention therapy; MO: *Moringa oleífera;* PG: *Punica granatum*; SB: *Scutellaria baicalensis*; SC: *Schisandra chinensis*. 16SrRNA:16S ribosomal ribonucleic acid (rRNA) sequencing; qPCR: quantitative polymerase chain reaction

**Table 3:** Anthropometric data and laboratory biomarkers of the studies included in the systematic review.

Study (N Intervention/N Control)	Intervention		Control		
Cao 2023 (18/19)	Change	SD	Change	SD	Mean difference [CI 95%]
Glucose	−0.17	0.74	0.05	0.78	−0.12 [−0.61, 0.37]
C-peptide	−0.5	1.23	0.1	1.33	−0.40 [−1.22, 0.42]
Insulin	−3.87	9.78	−2.1	13.33	−1.77 [−9.28, 5.74]
BMI	−1.31	1.1	−0.88	0.88	−0.43 [−1.05, 0.19]
Gomez-Martinez 2021 (31/34)	Change	SD	Change	SD	Mean difference [CI 95%]
Glucose	−2.80	7.8	2.0	13.2	−4.80 [−10.02, 0.42]
Insulin	1.26	4.02	1.82	4.24	−0.56 [−2.57, 1.45]
HbA1c	−0.09	0.30	0.04	0.34	−0.13 [−0.29, 0.03]
HOMA	0.24	1.06	0.57	1.4	−0.33 [−0.93, 0.27]
GLP	−0.80	4.93	−1.4	4.75	0.60 [−1.76, 2.96]
Ghrelin	−47.0	66.58	−42.6	65.48	−4.40 [−36.55, 27.75]
PYY	−6.0	17.08	−7.33	19.61	1.33 [−7.59, 10.25]
Shin 2019 (6/6)	Change	SD	Change	SD	Mean difference (CI 95%)
Glucose	3.2	4.66	5.3	4.51	−2.10 [−7.29, 3.09]
Insulin	0.58	0.52	0.72	0.87	−0.14 [−0.95, 0.67]
HbA1c	0.05	0.13	0.03	0.14	0.02 [−0.13, 0.17]
HOMA	0.21	0.13	0.29	0.26	−0.08 [−0.31, 0.15]
Weight	−0.05	1.72	0.46	1.65	−0.51 [−2.42, 1.40]
BMI	0.01	0.49	0.19	0.48	−0.18 [−0.73, 0.37]
Waist	−0.22	1.28	0.54	1.28	−0.76 [−2.21, 0.69]
Song 2015 (13/15)	Change	SD	Change	SD	Mean difference (CI 95%)
Glucose	−1.31	5.86	1.0	6.1	−2.31 [−6.75, 2.13]
Insulin	−0.41	4.26	−0.64	5.98	0.23 [−3.58, 4.04]
Cholesterol	−1.69	24.30	−5.6	20.74	3.91 [−12.96, 20.78]
HDL	−1.15	8.97	−8.4	20.42	7.25 [−4.18, 18.68]
TG	−27.46	109.8	11.6	45.84	−39.06 [−103.10, 24.98]
Weight	−0.54	12.25	−0.8	8.72	0.26 [−7.73, 8.25]
BMI	−0.2	3.42	−0.33	2.82	0.13 [−2.21, 2.47]
Waist	−1.88	6.81	−1.36	7.6	−0.52 [−5.86, 4.82]
%fat	−2.39	4.19	−1.35	3.11	−1.04 [−3.81, 1.73]

HbA1c: glycated haemoglobin; HOMA: homeostatic model assessment; GLP: glucagon-like peptide-1; PYY: peptide tyrosine tyrosine; HDL: high-density lipoproteins. TG: triglycerides; BMI: body mass index; SD: standard deviation; CI: confidence interval.

## Data Availability

Not applicable.

## Supplementary Materials

The following supporting information can be downloaded at: https://www.mdpi.com/article/10.3390/nu15092203/s1, Table S1: Search in Electronic databases.
