# Supplementary material for: Changes in the Gut Microbiota after the Use of Herbal Medicines in Overweight and Obese Individuals: A Systematic Review
Source: Nutrients. 2023 May 5;15(9):2203. doi: 10.3390/nu15092203 (PMC10181072; doi:10.3390/nu15092203)
Supplement: Supplementary file 1 [file nutrients-15-02203-s001.zip › nutrients-2307888-supplementary.pdf]

Suplementary Material 1. Search in Eletronic databases.

Medline via Ovid.

**Obesity**<br>Mesh Terms and	exp obesity/ or exp overweight/ or exp weight reduction programs/ or exp weight Loss/ or exp adiposity/ (obes* or overweight or over weight or overeat* or over eat* or adipos*).ti,ab. or ((bmi or body mass index or weight) adj2 (los* or change* or reduc*)).ti,ab<br>**AND**
**Gut Microbiota**	exp microbiota/ or exp gastrointestinal microbiome/ or exp dysbiosis or (Microbiota or microbiome or microflora or Gastrointestinal Flora or gut flora or intestinal flora or Gastrointestinal Microbial Communit* or enteric bacteria or pathogenic bacteria or beneficial bacteria or dysbiosis).ti,ab.<br><br>**AND**
**Herbal medicine**	exp herbal medicine/ or exp Phytotherapy/ or exp medicinal plant/ or exp Ethnopharmacology/ or exp Chinese herbal drugs/ or exp Plant Extracts/ or exp Traditional medicine/ or (herb* or phytotherapy or plant* or chinese medicine or traditional medicine).ti,ab. or ((folk or indigenous) adj3 (medicine or remed*)) or (Acacia meansii or Acanthopanax senticosus or Acanthopanax sessiliflorus or Actinidia arguta or Adiantum capillus-veneris or Aegle marmelos or Aesculus turbinate or Agave angustifolia or Agave potatorum or Aloe barbadensis or Aloe vera or Alpinia officinarum or Amorphophallus konjac or Araucaria angustifolia or Arum palaestinum or Aster yomena or Atractylodes lancea or Ba Qia or Benincasa hispida or Betula platyphylla or Black soya bean or blue berry or Bofu-tsusho-san or Bos indicus or Brassica nigra or Calabash or Calotropis procera Aiton or Cambogia or Camellia sinensis or Caper or Capparis decidua or Capparis sicula or Capsicum or Caralluma fimbriata or Caralluma quadrangular or Carissa carandas or Carthamus tinctorius or Cassia siamea or Catha edulis or Celastrus requelii or Chinese willow or Chrysanthemum indicum or Chuanshanlong or Cirsium setidens or Cissus quadrangular* or Citrus or Clusia nemroisa or Coffea arabica or Coleus forskohlii or Cordia salicifolia Cham or Corn silk or Cosmos caudatus or Crataegus azarolus or Crocus sativus or Cudrani1ricuspidateta or Curcuma longa or curcumim or Curry leaves or Cyclocarya paliurus or Cynara or Dioscorea or Diospyros kaki or Dunyeshuyu or Ecklonia cava or Eclipta alba or Eisenia bicyclis or Eleusine indica or Eugenia caryophyllus or Euphorbia supina or Evodiae or flos sophorae or fucoxanthin or Garcinia or Gardenia jasminoides or Gymnema or Ginkgo biloba or ginseng or Glucomannan or Glycine hispida or Glycine max or Glycyrrhiza uralensis or Glycyrrhizae or Griffonia simplicifolia or Guarana or Gymenma sylvestr* or Gypsum Fibrosum or Honeyberry or Ilex paraguariensis or Irvingia gabonenses or Kindal bark or Kokum fruit rind or Konjak or Kunth or Lagenaria siceraria or Ligularia fischeri or Limonia acidissima or Linggui Zhugan Decoction or Lonicera caerulea or Magnolia officinalis or Maidenhair fern or Malus hupehensis or

	Malus prunifolia or Malva parviflora or Matarique root or Maytenus ilicifolia Martius or Mondo grass or Morus alba or Morusaustrails poir or Murraya koenigii or Nelumbo nucifera or Nigella sativa or Niu-chang-chih or Omija or Ophiopogon japonicas or Salacia reticulat* or Origanum dayi or Oroxylum indicum or Paeoniae or Panax ginseng or Panax japonicas or Panax quinquefolium or Paullinia cupana or Perilla frutescens or Peucedanum japonicum Thunb or Phaseolus vulgaris or Platycodi or Platycodon grandiflorum or pomegranate seed oil or poria Macrocephalae or Prunus salicina or Psacalium decompositum or Psidium guajava or Punica granatum or Purple perilla or Radix or Ramulus Cinnamomi or Rhizoma coptidis or rhubarb or Coptis or Rhus coriaria or Rosmarinus officinalis or Salicornia europaea or Salix matsudana or Salvia officinalis or Sapindus rarak or Satiereal or Schisandra chinensis or Scutellariae or seme2ricusae or Sepiaria leaf or Sifangshuiniuji or Smilax china or Spirulina or Sojutsu or Solanum tuberosum or Sweet tea tree or Swertia chirayita or Swieteni2ricuspini or Silybum marianum or syriacum or talcum or Terminalia paniculate or Thunder god vine or Tripterygium wilfordii or Tuoshu or Turmeric or Ulam Raja or Vaccinium ashei or Vitis vinifera or Wasabia japonica Matsum or West African Plant or Withania somnifera or Xanthigen or Xin-ju-xiao-gaofang or Yellow pea fiber or Yerba mate or Zea mays or Zhemu fruit or Zicao or Zingiber officinale or GC or GNN).ti,ab<br>**AND**
**Study type**	(randomized controlled trial or clinical trial or controlled clinical trial).pt. or (random* or placebo or trial or group* or control* or crossover or parallel).ti,ab.<br>**NOT**
**Limits**	exp animals/ not humans.sh.

The Cochrane Central Register of Controlled Trials (CENTRAL)

**Obesity**	MeSH descriptor: [Obesity] explode all trees or MeSH descriptor: [Overweight] explode all trees or MeSH descriptor: [Weight Reduction Programs] explode all trees or MeSH descriptor: [Weight Loss] explode all trees MeSH descriptor: [Adiposity] explode all trees or (obes$ or overweight or over weight or overeat* or over eat* or adipos*):ti,ab,kw) or ((bmi or "body mass index" or weight) NEAR/2 (los* or change* or reduc*)):ti,ab,kw
	**AND**
**Gut Microbiota**	MeSH descriptor: [Microbiota] explode all trees or MeSH descriptor: [Gastrointestinal Microbiome] explode all trees MeSH descriptor: [Dysbiosis] explode all trees or (Microbiota or microbiome or microflora or "Gastrointestinal Flora" or "gut flora" or "intestinal flora" or "Gastrointestinal Microbial Communit*" or "enteric bacteria" or "pathogenic bacteria" or "beneficial bacteria" or dysbiosis):ti,ab,kw
	**AND**
**Herbal medicine**	MeSH descriptor: [Herbal Medicine] explode all trees or MeSH descriptor: [Phytotherapy] explode all trees or MeSH descriptor: [Plants, Medicinal] explode all trees or MeSH descriptor: [Chinese herbal drugs] explode all trees or MeSH descriptor: [Plant extracts] explode all trees or MeSH descriptor: [Ethnopharmacology] explode all trees or MeSH descriptor: [Traditional Medicine] explode all trees or (herb* or phytotherapy or plant* or “chinese medicine" or "traditional medicine"):ti,ab,kw or ((folk or indigenous) NEAR/2 (medicine or remed*)):ti,ab,kw or (“Acacia meansii” or “Acanthopanax senticosus” or “Acanthopanax sessiliflorus” or “Actinidia arguta” or “Adiantum capillus-veneris” or “Aegle marmelos” or “Aesculus turbinate” or “Agave angustifolia” or “Agave potatorum” or “Aloe barbadensis” or “Aloe vera” or “Alpinia officinarum” or “Amorphophallus konjac” or “Araucaria angustifolia” or “Arum palaestinum” or “Aster yomena” or “Atractylodes lancea” or “Ba Qia” or “Benincasa hispida” or “Betula platyphylla” or “Black soya bean” or “blue berry” or “Bofu-tsusho-san” or “Bos indicus” or “Brassica nigra” or Calabash or “Calotropis procera Aiton” or Cambogia or “Camellia sinensis” or Caper or “Capparis decidua” or “Capparis sicula” or Capsicum or “Caralluma fimbriate” or “Caralluma quadrangular” or “Carissa carandas” or “Carthamus tinctorius” or “Cassia siamea” or “Catha edulis” or “Celastrus requelii” or “Chinese willow” or “Chrysanthemum indicum” or Chuanshanlong or “Cirsium setidens” or “Cissus quadrangular*” or Citrus or “Clusia nemroisa” or “Coffea arabica” or “Coleus forskohlii” or “Cordia salicifolia Cham” or “Corn silk” or “Cosmos caudatus” or “Crataegus azarolus”

or “Crocus sativus” or “Cudrani4ricuspidateta” or “Curcuma longa” or curcumim or “Curry leaves” or “Cyclocarya paliurus” or Cynara or Dioscorea or “Diospyros kaki” or Dunyeshuyu or “Ecklonia cava” or “Eclipta alba” or “Eisenia bicyclis” or “Eleusine indica” or “Eugenia caryophyllus” or “Euphorbia supina” or Evodiae or “flos sophorae” or fucoxanthin or Garcinia or “Gardenia jasminoides” or Gymnema or “Ginkgo biloba” or ginseng or Glucomannan or “Glycine hispida” or “Glycine max” or “Glycyrrhiza uralensis” or Glycyrrhizae or “Griffonia simplicifolia” or Guarana or “Gymenma sylvestr$” or “Gypsum Fibrosum” or Honeyberry or “Ilex paraguariensis” or “Irvingia gabonenses” or “Kindal bark” or Kokum “fruit rind” or Konjak or Kunth or “Lagenaria siceraria” or “Ligularia fischeri” or “Limonia acidissima” or “Linggui Zhugan Decoction” or “Lonicera caerulea” or “Magnolia officinalis” or “Maidenhair fern” or “Malus hupehensis” or “Malus prunifolia” or “Malva parviflora” or “Matarique root” or “Maytenus ilicifolia Martius” or “Mondo grass” or “Morus alba” or “Morusaustrails poir” or “Murraya koenigii” or “Nelumbo nucifera” or “Nigella sativa” or Niu-chang-chih or Omija or “Ophiopogon japonicas” or “Salacia reticulat*” or “Origanum dayi” or “Oroxylum indicum” or Paeoniae or “Panax ginseng” or “Panax japonicas” or “Panax quinquefolium” or “Paullinia cupana” or “Perilla frutescens” or “Peucedanum japonicum Thunb” or “Phaseolus vulgaris” or Platycodi or “Platycodon grandiflorum” or “pomegranate seed oil” or “poria Macrocephalae” or “Prunus salicina” or “Psacalium decompositum” or “Psidium guajava” or “Punica granatum” or “Purple perilla” or Radix or “Ramulus Cinnamomi” or “Rhizoma coptidis” or rhubarb or Coptis or “Rhus coriaria” or “Rosmarinus officinalis” or “Salicornia europaea” or “Salix matsudana” or “Salvia officinalis” or “Sapindus rarak” or Satiereal or “Schisandra chinensis” or Scutellariae or “semen cassia” or “Sepiaria leaf” or Sifangshuiniuji or “Smilax china” or Spirulina or Sojutsu or “Solanum tuberosum” or “Sweet tea tree” or “Swertia chirayita” or “Swietenia mahogany” or “Silybum marianum” or syriacum or talcum or “Terminalia paniculate” or “Thunder god vine” or “Tripterygium wilfordii” or Tuoshu or Turmeric or “Ulam Raja” or “Vaccinium ashei” or “Vitis vinifera” or “Wasabia japonica Matsum” or “West African Plant” or “Withania somnifera” or Xanthigen or “Xin-ju-xiao-gaofang” or “Yellow pea fiber” or “Yerba mate” or “Zea mays” or “Zhemu fruit” or Zicao or “Zingiber officinale” or GC or GNN):ti,ab,kw

EMBASE

**Obesity'**	'obesity'/exp OR 'obesity' OR 'overweight'/exp OR 'overweight' OR 'weight reduction programs'/exp OR 'weight reduction programs' OR 'weight loss'/exp OR 'weight loss' OR 'adiposity'/exp OR 'adiposity' OR obes*:ti,ab OR overweight:ti,ab OR 'over weight':ti,ab OR overeat*:ti,ab OR 'over eat*':ti,ab OR adipos*:ti,ab ((bmi OR 'body mass index' OR weight) NEAR/2 (los* OR change* OR reduc*)):ti,ab
	**AND**
**Microbiot'**	'microbiota'/exp OR 'gastrointestinal microbiome'/exp OR dysbiosis/exp OR microbiota:ti,ab OR microbiome:ti,ab OR microflora:ti,ab OR 'gastrointestinal flora':ti,ab OR 'gut flora':ti,ab OR 'intestinal flora':ti,ab OR 'gastrointestinal microbial communit$':ti,ab OR 'enteric bacteria':ti,ab OR 'pathogenic bacteria':ti,ab OR 'beneficial bacteria':ti,ab OR dysbiosis:ti,ab
	**AND**
**Herbal medicine**	'herbal medicine'/exp OR 'phytotherapy'/exp OR 'medicinal plant'/exp OR Ethnopharmacology/exp OR 'Chinese herbal drugs'/exp OR 'Plant Extracts'/exp OR 'Traditional medicine'/exp OR herb*:ti,ab OR phytotherapy:ti,ab OR plant*:ti,ab OR 'chinese medicine':ti,ab OR 'traditional medicine':ti,ab OR ((folk or indigenous) NEAR/2 (medicine or remed$)):ti,ab OR'('acacia meansii':ti,ab OR 'acanthopanax senticosus':ti,ab OR 'acanthopanax sessiliflorus':ti,ab OR 'actinidia arguta':ti,ab OR 'adiantum capillus-veneris':ti,ab OR 'aegle marmelos':ti,ab OR 'aesculus turbinate':ti,ab OR 'agave angustifolia':ti,ab OR 'agave potatorum':ti,ab OR 'aloe barbadensis':ti,ab OR 'aloe vera':ti,ab OR 'alpinia officinarum':ti,ab OR 'amorphophallus konjac':ti,ab OR 'araucaria angustifolia':ti,ab OR 'arum palaestinum':ti,ab OR 'aster yomena':ti,ab OR 'atractylodes lancea':ti,ab OR 'ba qia':ti,ab OR 'benincasa hispida':ti,ab OR 'betula platyphylla':ti,ab OR 'black soya bean':ti,ab OR 'blue berry':ti,ab OR 'bofu-tsusho-san':ti,ab OR 'bos indicus':ti,ab OR 'brassica nigra':ti,ab OR calabash:ti,ab OR 'calotropis procera aiton':ti,ab OR cambogia:ti,ab OR 'camellia sinensis':ti,ab OR caper:ti,ab OR 'capparis decidua':ti,ab OR 'capparis sicula':ti,ab OR capsicum:ti,ab OR 'caralluma fimbriate':ti,ab OR 'caralluma quadrangular':ti,ab OR 'carissa carandas':ti,ab OR 'carthamus tinctorius':ti,ab OR 'cassia siamea':ti,ab OR 'catha edulis':ti,ab OR 'celastrus requelii':ti,ab OR 'chinese willow':ti,ab OR 'chrysanthemum indicum':ti,ab OR chuanshanlong:ti,ab OR 'cirsium setidens':ti,ab OR 'cissus quadrangular*':ti,ab OR citrus:ti,ab OR 'clusia nemroisa':ti,ab OR 'coffea arabica':ti,ab OR 'coleus forskohlii':ti,ab OR 'cordia salicifolia cham':ti,ab OR 'corn silk':ti,ab OR 'cosmos caudatus':ti,ab OR 'crataegus

azarolus':ti,ab OR 'crocus sativus':ti,ab OR 'cudrania tricuspidata':ti,ab OR 'curcuma longa':ti,ab OR curcumim:ti,ab OR 'curry leaves':ti,ab OR 'cyclocarya paliurus':ti,ab OR cynara:ti,ab OR dioscorea:ti,ab OR 'diospyros kaki':ti,ab OR dunyeshuyu:ti,ab OR 'ecklonia cava':ti,ab OR 'eclipta alba':ti,ab OR 'eisenia bicyclis':ti,ab OR 'eleusine indica':ti,ab OR 'eugenia caryophyllus':ti,ab OR 'euphorbia supina':ti,ab OR evodiae:ti,ab OR 'flos sophorae':ti,ab OR fucoxanthin:ti,ab OR garcinia:ti,ab OR 'gardenia jasminoides':ti,ab OR gymnema:ti,ab OR 'ginkgo biloba':ti,ab OR ginseng:ti,ab OR glucomannan:ti,ab OR 'glycine hispida':ti,ab OR 'glycine max':ti,ab OR 'glycyrrhiza uralensis':ti,ab OR glycyrrhizae:ti,ab OR 'griffonia simplicifolia':ti,ab OR guarana:ti,ab OR 'gymenma sylvestr*':ti,ab OR 'gypsum fibrosum':ti,ab OR honeyberry:ti,ab OR 'ilex paraguariensis':ti,ab OR 'irvingia gabonenses':ti,ab OR 'kindal bark':ti,ab OR kokum:ti,ab OR 'fruit rind':ti,ab OR konjak:ti,ab OR kunth:ti,ab OR 'lagenaria siceraria':ti,ab OR 'ligularia fischeri':ti,ab OR 'limonia acidissima':ti,ab OR 'linggui zhugan decoction':ti,ab OR 'lonicera caerulea':ti,ab OR 'magnolia officinalis':ti,ab OR 'maidenhair fern':ti,ab OR 'malus hupehensis':ti,ab OR 'malus prunifolia':ti,ab OR 'malva parviflora':ti,ab OR 'matarique root':ti,ab OR 'maytenus ilicifolia martius':ti,ab OR 'mondo grass':ti,ab OR 'morus alba':ti,ab OR 'morusaustrails poir':ti,ab OR 'murraya koenigii':ti,ab OR 'nelumbo nucifera':ti,ab OR 'nigella sativa':ti,ab OR 'niu chang chih':ti,ab OR omija:ti,ab OR 'ophiopogon japonicas':ti,ab OR ‘salacia reticulat$’:ti,ab OR 'origanum dayi':ti,ab OR 'oroxylum indicum':ti,ab OR paeoniae:ti,ab OR 'panax ginseng':ti,ab OR 'panax japonicas':ti,ab OR 'panax quinquefolium':ti,ab OR 'paullinia cupana':ti,ab OR 'perilla frutescens':ti,ab OR 'peucedanum japonicum thunb':ti,ab OR 'phaseolus vulgaris':ti,ab OR platycodi:ti,ab OR 'platycodon grandiflorum':ti,ab OR 'pomegranate seed oil':ti,ab OR 'poria macrocephalae':ti,ab OR 'prunus salicina':ti,ab OR 'psacalium decompositum':ti,ab OR 'psidium guajava':ti,ab OR 'punica granatum':ti,ab OR 'purple perilla':ti,ab OR radix:ti,ab OR 'ramulus cinnamomi':ti,ab OR 'rhizoma coptidis':ti,ab OR rhubarb:ti,ab OR coptis:ti,ab OR 'rhus coriaria':ti,ab OR 'rosmarinus officinalis':ti,ab OR 'salicornia europaea':ti,ab OR 'salix matsudana':ti,ab OR 'salvia officinalis':ti,ab OR 'sapindus rarak':ti,ab OR satiereal:ti,ab OR 'schisandra chinensis':ti,ab OR scutellariae:ti,ab OR 'semen cassia':ti,ab OR 'sepiaria leaf':ti,ab OR sifangshuiniuji:ti,ab OR 'smilax china':ti,ab OR spirulina:ti,ab OR sojutsu:ti,ab OR 'solanum tuberosum':ti,ab OR 'sweet tea tree':ti,ab OR 'swertia chirayita':ti,ab OR 'swietenia mahogany':ti,ab OR 'silybum marianum':ti,ab OR syriacum:ti,ab OR talcum:ti,ab OR 'terminalia paniculate':ti,ab OR 'thunder god vine':ti,ab OR 'tripterygium wilfordii':ti,ab OR tuoshu:ti,ab OR turmeric:ti,ab OR 'ulam raja':ti,ab OR 'vaccinium ashei':ti,ab OR 'vitis

	vinifera':ti,ab OR 'wasabia japonica matsum':ti,ab OR 'west african plant':ti,ab OR 'withania somnifera':ti,ab OR xanthigen:ti,ab OR 'xin-ju-xiao-gaofang':ti,ab OR 'yellow pea fiber':ti,ab OR 'yerba mate':ti,ab OR 'zea mays':ti,ab OR 'zhemu fruit':ti,ab OR zicao:ti,ab OR 'zingiber officinale':ti,ab OR gc:ti,ab OR gnn:ti,ab)
	**AND**
**Study type**	random*:ab,ti OR factorial*:ab,ti OR crossover*:ab,ti OR ((cross NEXT/1 over*):ab,ti) OR placebo*:ab,ti OR ((doubl* NEAR/1 blind*):ab,ti) OR ((singl* NEAR/1 blind*):ab,ti) OR assign*:ab,ti OR allocat*:ab,ti OR trial:ab,ti OR group*:ab,ti OR control*:ab,ti
	**NOT**
**Limits**	'animal'/exp OR 'nonhuman'/exp

Web of science (main collection of WEB of SCIENCE)

**Obesity**<br>Field Tag:<br>TOPIC	(obes* OR overweight OR “weight loss” OR “over weight” OR overeat* OR "over eat*") OR ((bmi OR "body mass index” OR weight) NEAR/2 (los* OR chang* OR reduc*))<br>**AND**
**Gut Microbiota**<br>Field Tag:<br>TOPIC	Microbiota OR microbiome OR microflora OR "Gastrointestinal Flora" OR "gut flora" OR "intestinal flora" OR "Gastrointestinal Microbial Communit*" OR "enteric bacteria" OR "pathogenic bacteria" OR "beneficial bacteria"<br>**AND**
**Herbal medicine**<br>Field Tag:<br>TOPIC	(herb* OR phytotherapy OR plant* OR chinese medicine" OR "traditional medicine" OR ((folk OR indigenous) NEAR/2 (medicine* OR remed*)) OR “Acacia meansii” OR “Acanthopanax senticosus” OR “Acanthopanax sessiliflorus” OR “Actinidia arguta” OR “Adiantum capillus-veneris” OR “Aegle marmelos” OR “Aesculus turbinate” OR “Agave angustifolia” OR “Agave potatorum” OR “Aloe barbadensis” OR “Aloe vera” OR “Alpinia officinarum” OR “Amorphophallus konjac” OR “Araucaria angustifolia” OR “Arum palaestinum” OR “Aster yomena” OR “Atractylodes lancea” OR “Ba Qia” OR “Benincasa hispida” OR “Betula platyphylla” OR “Black soya bean” OR “blue berry” OR “Bofu-tsusho-san” OR “Bos indicus” OR “Brassica nigra” OR Calabash OR “Calotropis procera Aiton” OR Cambogia OR “Camellia sinensis” OR Caper OR “Capparis decidua” OR “Capparis sicula” OR Capsicum OR “Caralluma fimbriate” OR “Caralluma quadrangular” OR “Carissa carandas” OR “Carthamus tinctorius” OR “Cassia siamea” OR “Catha edulis” OR “Celastrus requelii” OR “Chinese willow” OR “Chrysanthemum indicum” OR Chuanshanlong OR “Cirsium setidens” OR “Cissus quadrangular*” OR Citrus OR “Clusia nemroisa” OR “Coffea arabica” OR “Coleus forskohlii” OR “Cordia salicifolia Cham” OR “Corn silk” OR “Cosmos caudatus” OR “Crataegus azarolus” OR “Crocus sativus” OR “Cudrani8ricuspidateta” OR “Curcuma longa” OR curcumim OR “Curry leaves” OR “Cyclocarya paliurus” OR Cynara OR Dioscorea OR “Diospyros kaki” OR Dunyeshuyu OR “Ecklonia cava” OR “Eclipta alba” OR “Eisenia bicyclis” OR “Eleusine indica” OR “Eugenia caryophyllus” OR “Euphorbia supina” OR Evodiae OR “flos sophorae” OR fucoxanthin OR Garcinia OR “Gardenia jasminoides” OR Gymnema OR “Ginkgo biloba” OR ginseng OR Glucomannan OR “Glycine hispida” OR “Glycine max” OR “Glycyrrhiza uralensis” OR Glycyrrhizae OR “Griffonia simplicifolia” OR Guarana OR “Gymenma sylvestr$” OR “Gypsum Fibrosum” OR Honeyberry OR “Ilex paraguariensis” OR “Irvingia gabonenses” OR “Kindal bark” OR Kokum “fruit rind” OR Konjak OR Kunth OR “Lagenaria siceraria” OR “Ligularia fischeri” OR “Limonia acidissima” OR “Linggui Zhugan Decoction” OR “Lonicera caerulea” OR “Magnolia officinalis”

	OR “Maidenhair fern” OR “Malus hupehensis” OR “Malus prunifolia” OR “Malva parviflora” OR “Matarique root” OR “Maytenus ilicifolia Martius” OR “Mondo grass” OR “Morus alba” OR “Morusaustrails poir” OR “Murraya koenigii” OR “Nelumbo nucifera” OR “Nigella sativa” OR Niu-chang-chih OR Omija OR “Ophiopogon japonicas” OR “Salacia reticulat*” OR “Origanum dayi” OR “Oroxylum indicum” OR Paeoniae OR “Panax ginseng” OR “Panax japonicas” OR “Panax quinquefolium” OR “Paullinia cupana” OR “Perilla frutescens” OR “Peucedanum japonicum Thunb” OR “Phaseolus vulgaris” OR Platycodi OR “Platycodon grandiflorum” OR “pomegranate seed oil” OR “poria Macrocephalae” OR “Prunus salicina” OR “Psacalium decompositum” OR “Psidium guajava” OR “Punica granatum” OR “Purple perilla” OR Radix OR “Ramulus Cinnamomi” OR “Rhizoma coptidis” OR rhubarb OR Coptis OR “Rhus coriaria” OR “Rosmarinus officinalis” OR “Salicornia europaea” OR “Salix matsudana” OR “Salvia officinalis” OR “Sapindus rarak” OR Satiereal OR “Schisandra chinensis” OR Scutellariae OR “semen cassia” OR “Sepiaria leaf” OR Sifangshuiniuji OR “Smilax china” OR Spirulina OR Sojutsu OR “Solanum tuberosum” OR “Sweet tea tree” OR “Swertia chirayita” OR “Swietenia mahogany” OR “Silybum marianum” OR syriacum OR talcum OR “Terminalia paniculate” OR “Thunder god vine” OR “Tripterygium wilfORdii” OR Tuoshu OR Turmeric OR “Ulam Raja” OR “Vaccinium ashei” OR “Vitis vinifera” OR “Wasabia japonica Matsum” OR “West African Plant” OR “Withania somnifera” OR Xanthigen OR “Xin-ju-xiao-gaofang” OR “Yellow pea fiber” OR “Yerba mate” OR “Zea mays” OR “Zhemu fruit” OR Zicao OR “Zingiber officinale” OR GC OR GNN)<br>**NOT**
**Limit**<br>Field Tag: Abstract	AB=(animal* OR mice OR rat* OR mouse)

Search in Scopus

**Obesity**<br>Field Tag: abstract, title and key-words	(TITLE-ABS-KEY (obes* OR overweight OR “weight loss” OR “over weight” OR overeat* OR "over eat*" OR adipos*) OR ((bmi O“ "body mass index” OR weight) W/2 (los* OR change* OR reduc*)))<br>**AND**
**Gut Microbiota**<br>Field Tag: abstract, title and key-words	(TITLE-ABS-KEY (Microbiota OR “Gastrointestinal Microbiome” OR Microbiota OR microbiome OR microflora OR "Gastrointestinal Flora" OR "gut flora" OR "intestinal flora" OR "Gastrointestinal Microbial Communit*" OR "enteric bacteria" OR "pathogenic bacteria" OR "beneficial bacteria" OR dysbiosis))<br>**AND**
**Herbal medicine**<br>Field Tag: abstract, title and key-words	(TITLE-ABS-KEY (herb* OR phytotherapy OR plant* OR chinese medicine" OR "traditional medicine" OR ((folk OR indigenous) W/2 (medicine* OR remed*)) OR “Acacia meansii” OR “Acanthopanax senticosus” OR “Acanthopanax sessiliflORus” OR “Actinidia arguta” OR “Adiantum capillus-veneris” OR “Aegle marmelos” OR “Aesculus turbinate” OR “Agave angustifolia” OR “Agave potatorum” OR “Aloe barbadensis” OR “Aloe vera” OR “Alpinia officinarum” OR “Amorphophallus konjac” OR “Araucaria angustifolia” OR “Arum palaestinum” OR “Aster yomena” OR “Atractylodes lancea” OR “Ba Qia” OR “Benincasa hispida” OR “Betula platyphylla” OR “Black soya bean” OR “blue berry” OR “Bofu-tsusho-san” OR “Bos indicus” OR “Brassica nigra” OR Calabash OR “Calotropis procera Aiton” OR Cambogia OR “Camellia sinensis” OR Caper OR “Capparis decidua” OR “Capparis sicula” OR Capsicum OR “Caralluma fimbriate” OR “Caralluma quadrangular” OR “Carissa carandas” OR “Carthamus tinctorius” OR “Cassia siamea” OR “Catha edulis” OR “Celastrus requelii” OR “Chinese willow” OR “Chrysanthemum indicum” OR Chuanshanlong OR “Cirsium setidens” OR “Cissus quadrangular*” OR Citrus OR “Clusia nemroisa” OR “Coffea arabica” OR “Coleus forskohlii” OR “Cordia salicifolia Cham” OR “Corn silk” OR “Cosmos caudatus” OR “Crataegus azarolus” OR “Crocus sativus” OR “Cudrani10ricuspidateta” OR “Curcuma longa” OR curcumim OR “Curry leaves” OR “Cyclocarya paliurus” OR Cynara OR Dioscorea OR “Diospyros kaki” OR Dunyeshuyu OR “Ecklonia cava” OR “Eclipta alba” OR “Eisenia bicyclis” OR “Eleusine indica” OR “Eugenia caryophyllus” OR “Euphorbia supina” OR Evodiae OR “flos sophorae” OR fucoxanthin OR Garcinia OR “Gardenia jasminoides” OR Gymnema OR “Ginkgo biloba” OR ginseng OR Glucomannan OR “Glycine hispida” OR “Glycine max” OR “Glycyrrhiza uralensis” OR Glycyrrhizae OR “Griffonia simplicifolia” OR Guarana OR “Gymenma sylvestr$” OR “Gypsum Fibrosum” OR Honeyberry OR “Ilex paraguariensis” OR “Irvingia gabonenses” OR “Kindal bark” OR Kokum “fruit rind” OR Konjak OR Kunth OR “Lagenaria siceraria” OR “Ligularia fischeri” OR “Limonia acidissima” OR “Linggui Zhugan Decoction” OR “Lonicera caerulea” OR “Magnolia officinalis” OR “Maidenhair fern” OR “Malus hupehensis” OR “Malus prunifolia” OR “Malva

parviflora” OR “Matarique root” OR “Maytenus ilicifolia Martius” OR “Mondo grass” OR “Morus alba” OR “Morusaustrails poir” OR “Murraya koenigii” OR “Nelumbo nucifera” OR “Nigella sativa” OR Niu-chang-chih OR Omija OR “Ophiopogon japonicas” OR “Salacia reticulat*” OR “Origanum dayi” OR “Oroxylum indicum” OR Paeoniae OR “Panax ginseng” OR “Panax japonicas” OR “Panax quinquefolium” OR “Paullinia cupana” OR “Perilla frutescens” OR “Peucedanum japonicum Thunb” OR “Phaseolus vulgaris” OR Platycodi OR “Platycodon grandiflorum” OR “pomegranate seed oil” OR “poria Macrocephalae” OR “Prunus salicina” OR “Psacalium decompositum” OR “Psidium guajava” OR “Punica granatum” OR “Purple perilla” OR Radix OR “Ramulus Cinnamomi” OR “Rhizoma coptidis” OR rhubarb OR Coptis OR “Rhus coriaria” OR “Rosmarinus officinalis” OR “Salicornia europaea” OR “Salix matsudana” OR “Salvia officinalis” OR “Sapindus rarak” OR Satiereal OR “Schisandra chinensis” OR Scutellariae OR “semen cassia” OR “Sepiaria leaf” OR Sifangshuiniuji OR “Smilax china” OR Spirulina OR Sojutsu OR “Solanum tuberosum” OR “Sweet tea tree” OR “Swertia chirayita” OR “Swietenia mahogany” OR “Silybum marianum” OR syriacum OR talcum OR “Terminalia paniculate” OR “Thunder god vine” OR “Tripterygium wilfORdii” OR Tuoshu OR Turmeric OR “Ulam Raja” OR “Vaccinium ashei” OR “Vitis vinifera” OR “Wasabia japonica Matsum” OR “West African Plant” OR “Withania somnifera” OR Xanthigen OR “Xin-ju-xiao-gaofang” OR “Yellow pea fiber” OR “Yerba mate” OR “Zea mays” OR “Zhemu fruit” OR Zicao OR “Zingiber officinale” OR GC OR GNN))
